# RelA NF-κB subunit activation as a therapeutic target in diffuse large B-cell lymphoma

**DOI:** 10.18632/aging.101121

**Published:** 2016-12-08

**Authors:** Mingzhi Zhang, Zijun Y. Xu-Monette, Ling Li, Ganiraju C. Manyam, Carlo Visco, Alexandar Tzankov, Jing Wang, Santiago Montes-Moreno, Karen Dybkaer, April Chiu, Attilio Orazi, Youli Zu, Govind Bhagat, Kristy L. Richards, Eric D. Hsi, William W.L. Choi, J. Han van Krieken, Jooryung Huh, Maurilio Ponzoni, Andrés J.M. Ferreri, Michael B. Møller, Ben M. Parsons, Jane N. Winter, Miguel A. Piris, L. Jeffrey Medeiros, Lan V. Pham, Ken H. Young

**Affiliations:** ^1^ Department of Oncology, The First Affiliated Hospital Zhengzhou University, Zhengzhou, Henan, China; ^2^ Department of Hematopathology, The University of Texas MD Anderson Cancer Center, Houston, TX 77030, USA; ^3^ Department of Bioinformatics and Computational Biology, The University of Texas MD Anderson Cancer Center, Houston, TX 77030, USA; ^4^ San Bortolo Hospital, Vicenza, Italy; ^5^ University Hospital, Basel, Switzerland; ^6^ Hospital Universitario Marques de Valdecilla, Santander, Spain; ^7^ Aalborg University Hospital, Aalborg, Denmark; ^8^ Memorial Sloan-Kettering Cancer Center, New York, NY 10065, USA; ^9^ Weill Medical College of Cornell University, New York, NY 10065, USA; ^10^ The Methodist Hospital, Houston, TX 77030, USA; ^11^ Columbia University Medical Center and New York Presbyterian Hospital, New York, NY 10032, USA; ^12^ University of North Carolina School of Medicine, Chapel Hill, NC 27514, USA; ^13^ Cleveland Clinic, Cleveland, OH 44195, USA; ^14^ University of Hong Kong Li Ka Shing Faculty of Medicine, Hong Kong, China; ^15^ Radboud University Nijmegen Medical Centre, Nijmegen, the Netherlands; ^16^ Asan Medical Center, Ulsan University College of Medicine, Seoul, Korea; ^17^ San Raffaele H. Scientific Institute, Milan, Italy; ^18^ Odense University Hospital, Odense, Denmark; ^19^ Gundersen Medical Foundation, La Crosse, WI 54601, USA; ^20^ Feinberg School of Medicine, Northwestern University, Chicago, IL 60611, USA; ^21^ The University of Texas School of Medicine, Graduate School of Biomedical Sciences, Houston, TX 77030, USA

**Keywords:** NF-κB, p65, diffuse large B-cell lymphoma, TP53, GCB, gene expression profiling, proteasome inhibitor

## Abstract

It has been well established that nuclear factor kappa-B (NF-κB) activation is important for tumor cell growth and survival. RelA/p65 and p50 are the most common NF-κB subunits and involved in the classical NF-κB pathway. However, the prognostic and biological significance of RelA/p65 is equivocal in the field. In this study, we assessed RelA/p65 nuclear expression by immunohistochemistry in 487 patients with *de novo* diffuse large B-cell lymphoma (DLBCL), and studied the effects of molecular and pharmacological inhibition of NF-κB on cell viability. We found RelA/p65 nuclear expression, without associations with other apparent genetic or phenotypic abnormalities, had unfavorable prognostic impact in patients with stage I/II DLBCL. Gene expressionprofiling analysis suggested immune dysregulation and antiapoptosis may be relevant for the poorer prognosis associated with p65 hyperactivation in germinal center B-cell–like (GCB) DLBCL and in activated B-cell–like (ABC) DLBCL, respectively. We knocked down individual NF-κB subunits in representative DLBCL cells in vitro, and found targeting p65 was more effective than targeting other NF-κB subunits in inhibiting cell growth and survival. In summary, RelA/p65 nuclear overexpression correlates with significant poor survival in early-stage DLBCL patients, and therapeutic targeting RelA/p65 is effective in inhibiting proliferation and survival of DLBCL with NF-κB hyperactivation.

## INTRODUCTION

Diffuse large B-cell lymphoma (DLBCL), the most common form of aggressive non-Hodgkin lymphoma, accounts for nearly 40% of non-Hodgkin lymphomas [1]. Although most cases of DLBCL are curable with the standard immunochemotherapy regimen, rituximab plus cyclophosphamide, hydroxydaunomycin, vincris-tine, and prednisone (R-CHOP), 30-40% of patients have drug-resistant disease or recurrence [2]. DLBCL is a highly heterogeneous disease. Based on gene expression profiling (GEP), DLBCL can be classified into two molecular subtypes: germinal center B-cell–like (GCB) and activated B-cell–like (ABC) DLBCL [3]. The ABC subtype of DLBCL typically exhibits constitutive activation of the nuclear factor-kappaB (NF-κB) pathway [4, 5] and patients have inferior clinical outcomes compared with patients with GCB-DLBCL [6, 7]. Recent studies have shown that NF-κB expression is not limited to ABC-DLBCL but also can occur in GCB-DLBCL [8-10].

The NF-κB/Rel family contains five transcription factors: RelA (p65), NF-κB1 (p50; p105), NF-κB2 (p52; p100), RelB, and c-Rel. Only RelA/p65, RelB, and c-Rel had transactivation domains [11]. NF-κB activity is controlled by inhibitors of NF-κB (such as IκBα which inhibits p65/p50 dimers) that keep NF-κB inactive in the cytoplasm. Constitutive activation of NF-κB in ABC-DLBCL is caused by chronic activation of B-cell-receptor (BCR) signaling and elevated IκB kinase (IKK) activities which phosphorylate IκBα. As a result, IκBα is degraded releasing homo- or hetero-dimers of NF-κB to enter the nucleus where NF-κB activates gene transcription [4, 12-14]. *In vivo* the most abundant NF-κB dimers are p50/p65 heterodimers which are ubiquitously expressed in mammalian tissue [11, 15-17], consistent with the highest level of nuclear p50/p65 in DLBCL samples among all NF-κB subunits by our previous studies [10, 18]. Detection of p65/p50 nuclear expression in tumor cells has been considered as a surrogate marker of NF-κB activation through the canonical pathway [9]. p65 also can form p65/p65 homodimers with distinct DNA-binding modes and functions [19-21].

NF-κB activation suppresses apoptosis and promotes tumor cell survival and proliferation, leading to treatment resistance. Different NF-κB subunits had distinct and overlapping functions [22-24]. In addition, transcriptional and functional crosstalk between antiapoptotic NF-κB and proapoptotic p53 (an essential tumor suppressor) plays a critical role in determining the fate of tumor cells [25, 26]. The p65 subunit of NF-κB and p53 counteract each other's function in regulating cell proliferation, metabolism and apoptosis [25, 27-29]. p65 increases MDM2 levels, which decrease the stabilization of p53 and cell death induced by cytotoxic chemotherapy [25]. However, cooperation between p65 and p53 has been also reported [30-33], making interactions between p65/NF-κB and p53 much more complicated. Both p53 and p65 were unexpectedly found necessary for either p53 or NF-κB-directed gene transcription under replicational stress or atypical and classical stimuli for NF-κB. Induced p65 in stimulated cancer cells by pro-inflammatory tumor necrosis factor α (TNF-α) binds to p53 and the p65/p53 complex transcriptionally activates NF-κB target genes (*survivin/BIRC5*, *BCL2*, *BCL-XL*, and *FASL*) [32]. Moreover, p65 and p53 co-regulate induction of proinflammatory genes in monocytes and macrophages [33].

Despite the well-established role of NF-κB signaling in lymphoma pathogenesis and treatment resistance, conflicting results on the prognostic significance of NF-κB and RelA/p65 expression (as a surrogate marker of NF-κB activation) in DLBCL have been reported by previous clinical studies [8, 9, 34-36]. To help clarify the prognostic effect of RelA/p65 nuclear expression, in this study we evaluated nuclear expression of RelA/p65 by immunohistochemistry (IHC) in a large cohort of DLBCL treated with R-CHOP, and studied the prog-nostic effects and gene expression profiles associated with p65 nuclear expression. Moreover, we inactivated individual NF-κB subunits in vitro and investigated their differential effects on proliferation and apoptosis of DLBCL cells which highlighted the important therapeutic value of RelA/p65.

## RESULTS

### p65 hyperactivation has significant adverse impact in early-stage DLBCL

**Figure 1:**
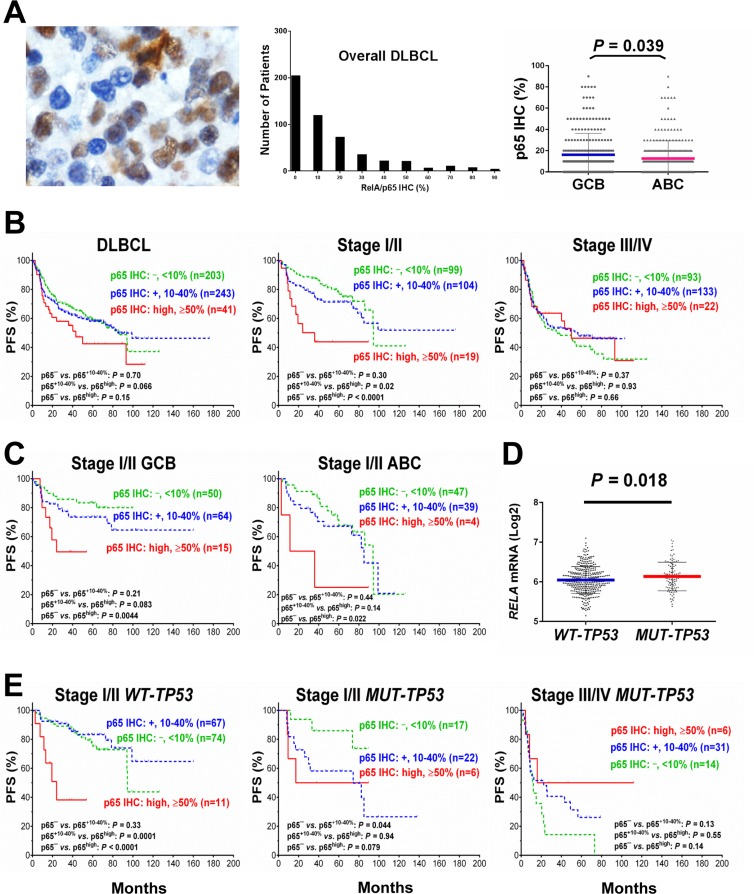
Nuclear expression of p65 and its effect on progression-free survival (PFS) in diffuse large B-cell lymphoma (DLBCL) (**A**) Representative immunohistochemical analysis (IHC) and histograms for p65 nuclear expression in DLBCL. The mean expression of nuclear p65 was significantly higher in the germinal center B-cell–like (GCB) subtype than in the activated B-cell–like (ABC) subtype. (**B**) In overall DLBCL, high p65 nuclear expression (p65^high^, ≥50% nuclear expression) was associated with a trend towards worse PFS. In patients with stage I/II DLBCL, p65^high^ correlated with significantly shorter PFS. In patients with stage III/IV DLBCL, p65^high^ did not show signficant prognostic impact. (**C**) p65^high^ correlate with significantly shorter PFS in patients with stage I/II DLBCL independent of GCB/ABC subtypes. (**D**) *TP53* mutation status was significantly associated with higher *RELA* mRNA expression. (**E**) In patients with stage I/II DLBCL, p65^high^ correlate with significantly shorter PFS independent of *TP53* mutation status although more significant in patients with wild-type *TP*53 (*WT-TP53*). In patients with mutated *TP53* (*MUT-TP53*) and stage III/IV DLBCL, p65^high^ was associated with a trend of better PFS.

p65 expression was evaluable in 487 DLBCL patients, including 287 men and 200 women. GCB/ABC ratio was close to 1 (243 GCB and 239 ABC). The median age of the patients in the study group was 63 years, and 58% of the study cohort had elderly age (≥60). Immunohistochemical results showed that 58% of DLBCL samples had p65 nuclear expression indicative of p65 activation at various levels (Fig. 1A) with a mean level of 14%. p65 nuclear expression was not specific for ABC-DLBCL. In fact, the GCB-DLBCL group had a slightly higher mean level of p65 nuclear expression (16.1%) than the ABC-DLBC group (12.6%) (Fig. 1A). Table 1 showed the clinical and pathological features of the study cohort.

**Table 1:** Clinical characteristics of 487 patients with *de novo* diffuse large B-cell lymphoma (DLBCL)

	DLBCL			GCB-DLBCL			ABC-DLBCL			*WT-TP53*			*MUT-TP53*		
	p65^high^	p65^low^	*P*	p65^high^	p65^low^	*P*	p65^high^	p65^low^	*P*	p65^high^	p65^low^	*P*	p65^high^	p65^low^	*P*
Characteristics	N (%)	N (%)		N (%)	N (%)		N (%)	N (%)		N (%)	N (%)		N (%)	N (%)	
**Patients**	41	446		28	215		13	226		26	312		12	84	
**Age (years)**															
<60	21 (51)	183 (41)	0.21	16 (57)	106 (49)	0.44	5 (39)	74 (33)	0.67	13 (50)	124 (40)	0.31	7 (58)	30 (36)	0.13
≥60	20 (49)	263 (59)		12 (43)	109 (51)		8 (61)	152 (67)		13 (50)	188 (60)		5 (42)	54 (64)	
**Gender**															
Female	8 (20)	192 (43)	**0.003**	6 (21)	95 (44)	**0.022**	2 (15)	95 (42)	0.057	6 (30)	130 (42)	0.063	2 (17)	38 (45)	0.06
Male	33 (80)	254 (57)		22 (79)	120 (56)		11 (85)	131 (58)		20 (70)	182 (58)		10 (83)	46 (55)	
**Stage**															
I/II	19 (46)	203 (47)	0.90	15 (54)	114 (56)	0.84	4 (31)	86 (39)	0.54	11 (42)	141 (48)	0.60	6 (50)	39 (46)	0.82
III/IV	22 (54)	226 (53)		13 (46)	91 (45)		9 (69)	133 (61)		15 (58)	155 (52)		6 (50)	45 (54)	
**B-symptoms**															
No	25 (61)	272 (64)	0.67	18 (64)	141 (70)	0.55	7 (54)	127 (59)	0.73	16 (61)	196 (66)	0.61	7 (58)	54 (68)	0.53
Yes	16 (39)	151 (36)		10 (36)	61 (30)		6 (46)	89 (41)		10 (39)	99 (34)		5 (42)	26 (33)	
**LDH levels**															
Normal	14 (34)	161 (40)	0.51	8 (29)	86 (44)	0.12	6 (46)	74 (36)	0.44	10 (39)	121 (43)	0.65	3 (25)	28 (36)	0.46
Elevated	27 (66)	247 (60)		20 (71)	109 (56)		7 (54)	134 (64)		16 (61)	160 (57)		9 (75)	50 (64)	
**Extranodal sites (n)**															
0–1	35 (85)	327 (77)	0.22	23 (82)	160 (80)	0.75	12 (92)	163 (74)	0.15	21 (81)	231 (79)	0.79	11 (92)	64 (78)	0.27
≥2	6 (15)	98 (23)		5 (18)	41 (20)		1 (8)	56 (26)		5 (19)	63 (21)		1 (8)	18 (22)	
**Performance status**															
0–1	34 (87)	329 (83)	0.53	23 (85)	158 (86)	0.87	11 (92)	166 (80)	0.33	22 (85)	231 (85)	0.93	10 (91)	69 (90)	0.89
≥2	5 (13)	66 (17)		4 (15)	25 (14)		1 (8)	41 (20)		4 (15)	40 (15)		1 (9)	8 (10)	
**Size of largest tumor**															
<5cm	14 (44)	192 (58)	0.11	8 (35)	97 (63)	**0.011**	6 (67)	93 (54)	0.47	8 (38)	146 (62)	**0.035**	5 (50)	33 (49)	0.96
≥5cm	18 (56)	137 (42)		15 (65)	58 (37)		3 (33)	78 (46)		13 (62)	91 (38)		5 (50)	34 (51)	
**IPI risk group**															
0–2	29 (71)	267 (62)	0.25	21 (75)	144 (71)	0.82	8 (61)	118 (54)	0.78	17 (65)	189 (63)	1.0	10 (83)	47 (57)	0.12
3-5	12 (29)	162 (38)		7 (25)	60 (29)		5 (39)	102 (46)		9 (35)	109 (37)		2 (17)	43 (35)	
**Therapy response**															
CR	29 (71)	343 (77)	0.37	17 (61)	166 (77)	0.057	12 (92)	172 (76)	0.18	17 (65)	257 (82)	**0.034**	9 (75)	49 (58)	0.27
Non-CR	12 (29)	103 (23)		11 (39)	49 (23)		1 (8)	22 (24)		9 (35)	55 (18)		3 (25)	35 (42)	
**Primary origin**															
Extranodal	20 (49)	149 (34)	0.058	14 (50)	68 (32)	0.063	6 (46)	79 (36)	0.44	14 (54)	102 (33)	**0.035**	4 (33)	26 (32)	0.91
Nodal	21 (51)	289 (66)		14 (50)	143 (68)		7 (54)	143 (64)		12 (46)	204 (67)		8 (67)	56 (68)	
**Cell-of-origin**															
GCB	28 (68)	215 (49)	**0.022**	-	-	-	-	-	**-**	18 (69)	143 (46)	**0.039**	9 (75)	49 (58)	0.35
ABC	13 (32)	226 (51)		-	-		-	-		8 (31)	165 (54)		3 (25)	35 (42)	
**p50 nuclear expression**															
<20%	15 (40)	278 (67)	**0.002**	12 (46)	149 (74)	**0.005**	3 (27)	129 (60)	**0.05**	9 (41)	188 (65)	**0.037**	6 (50)	57 (71)	0.18
≥20%	22 (60)	138 (33)		14 (54)	52 (26)		8 (73)	85 (40)		13 (59)	102 (35)		6 (50)	23 (29)	
**p52 nuclear expression**															
–	21 (55)	300 (71)	**0.043**	14 (54)	142 (71)	0.11	7 (58)	157 (72)	0.33	14 (58)	208 (71)	0.25	5 (46)	59 (74)	0.078
+	17 (45)	120 (29)		12 (46)	58 (29)		5 (42)	61 (28)		10 (42)	86 (29)		6 (54)	21 (26)	
**c-Rel nuclear expression**															
–	17 (46)	297 (72)	**0.002**	11 (44)	147 (74)	**0.004**	6 (50)	150 (70)	0.20	9 (41)	207 (73)	**0.003**	7 (58)	55 (67)	0.53
+	20 (54)	117 (28)		14 (56)	52 (26)		6 (50)	64 (30)		13 (59)	78 (27)		5 (42)	27 (33)	
**Bcl-2 expression**															
<70%	27 (66)	229 (52)	0.10	23 (82)	127 (60)	**0.036**	4 (31)	99 (44)	0.40	17 (65)	164 (53)	0.31	7 (58)	40 (48)	0.55
≥70%	14 (34)	208 (48)		5 (18)	83 (40)		9 (69)	125 (56)		9 (35)	143 (47)		5 (42)	44 (52)	

Abbreviations: p65^high^, high levels of nuclear p65; p65^low^, low levels of p65 nuclear expression; LDH, lactate dehydrogenase; IPI, international prognostic index; CR, complete remission; PR, partial response; GCB, germinal center B-cell–like; ABC, activated B-cell–like; *WT-TP53*, wild-type *TP53*; *MUT-TP53*, mutated *TP53*. * Some of the clinicopathologic data were not available. Percentages are calculated among cases with specific data available. Significant *P* values in bold.

**Figure 2:**
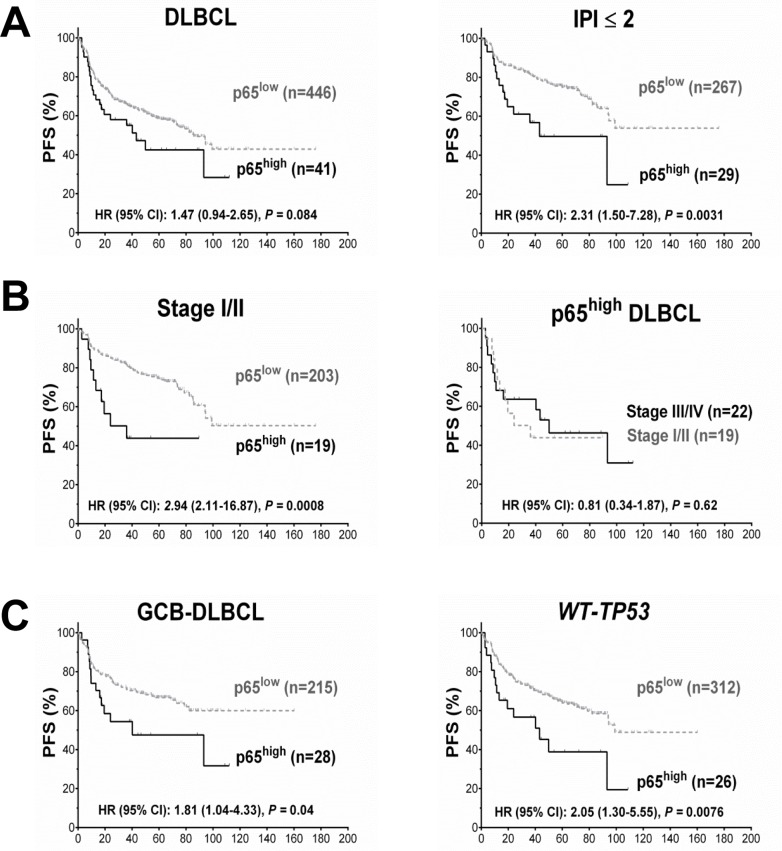
Prognosis for p65 hyperactivation in diffuse large B-cell lymphoma (DLBCL) (**A**) In overall DLBCL, high p65 nuclear expression (p65^high^, ≥50% nuclear expression) was associated with unfavorable progression-free survival (PFS). The adverse prognostic impact was significant in patients with an international prognostic index score (IPI) ≤2. (**B**) In patients with stage I/II DLBCL, p65^high^ correlated with significantly poorer PFS. Among p65^high^ DLBCL patients, disease stages did not show further prognostic impact. (**C**) p65^high^ correlated with significantly poorer PFS in patients with GCB-DLBCL and patients with wild-type *TP*53 (*WT-TP53*).

Low levels (10-40%) of p65 nuclear expression did not have significant prognostic impact in DLBCL (Fig. 1B). However, high p65 nuclear expression (p65^high^, ≥50% tumor cells with p65 positive nuclei) correlated with significantly shorter PFS and OS durations in patients with stage I/II DLBCL and in patients with an International Prognostic Index score (IPI) ≤2 (Fig. 1B, Fig. 2A). In contrast, in patients with stage III/IV DLBCL or an IPI >2, p65 expression was not prognostic. p65^high^ patients with stage I/II DLBCL had similar survival rates compared with p65^high^ patients with stage III/IV DLBCL (Fig. 2B).

When analyzed individually in GCB and ABC subtypes, in GCB-DLBCL only, the p65^high^ group frequently had large (≥ 5cm) tumors (65% *vs*. 37%, *P* = 0.011) (Table 1), and significantly decreased PFS (*P* = 0.04, Fig. 2C) and OS (*P* = 0.015) rates than other patients (p65^low^ group, IHC <50%). However, the unfavorable prognostic effect manifested in GCB-DLBCL was limited in stage I/II (Fig. 1C) and minimal in stage III/IV GCB-DLBCL (*P* = 0.95 for PFS and *P* = 0.60 for OS); also, in stage I/II ABC-DLBCL patients, p65^high^ expression also significantly correlated with worse PFS (Fig. 1C).

### p65 nuclear expression correlates with p50 nuclear expression in DLBCL

We found high p65 nuclear expression was significantly associated with p50^+^ and p50^high^ nuclear expression in overall DLBCL, GCB-DLBCL, and ABC-DLBCL (Table 1), suggesting the predominance of p65/p50 dimer activation via the canonical NF-κB pathway [9]. Significant association with c-Rel^+^ nuclear expression was also found in overall DLBCL and GCB-DLBCL (p50/c-Rel is another dimer activated via the canonical pathway [37, 38]). No significant association was observed between p65^high^ and RelB^+^. p65^high^ showed significant association with p52^+^ in overall DLBCL but not in either GCB or ABC subset.

Nuclear expression of p50, p52, and c-Rel did not show further prognostic effects among the p65^high^ patients. We did not observe associations of p65^high^ with any other adverse biomarkers such as *TP53* mutations, *MYC*/*BCL2* translocation, and Myc/Bcl-2 over-expression which may confound the prognostic effects [39-42]. In contrast, in the GCB but not the ABC subgroup, p65^high^ compared with p65^low^ patients less frequently had Bcl-2 overexpression (18% *vs*. 40%, *P* = 0.036).

### p65 hyperactivation has significant adverse impact in patients with wild-type *TP53*

Cases of DLBCL with wild-type *TP53* (*WT-TP53*) had significantly lower levels of *RELA* mRNA (*P* = 0.018, Fig. 1D) and a trend toward lower nuclear p65 levels (*P* = 0.11) than those with mutated *TP53* (*MUT-TP53*), suggesting that wild-type p53 suppressed *RELA* NF-κB expression. Conversely, p65 antagonized p53 function as suggested by survival analysis: in *WT-TP53* DLBCL, patients with p65^high^ expression correlated with significantly decreased PFS (*P* = 0.0076, Fig. 2C) and OS (*P* = 0.0082) rates than patients with p65^low^ tumors, independent of GCB and ABC cell-of-origin. However, when subdivided cohorts by disease stages, we found the prognostic impact was only significant in patients with stage I/II disease (*P* < 0.0001 for PFS, *P* = 0.0004 for OS). Also in *MUT-TP53* patients with stage I/II DLBCL, positive p65 nuclear expression was associated with significant poorer survival; in contrast, opposite trends were observed in *MUT-TP53* patients with stage III/IV DLBCL (Fig. 1E).

### Multivariate survival analysis

Multivariate survival analysis (Cox regression) for high p65 nuclear expression with adjustments for clinical variables confirmed that p65^high^ was an independent adverse prognostic factor in patients with GCB-DLBCL and in patients with *WT-TP53* DLBCL, but not in the overall study group, the ABC-DLBCL subgroup, or the *MUT-TP53* DLBCL subgroup (Table 2).

**Table 2:** Multivariate analysis of clinicopathologic parameters for survival of patients with diffuse large B-cell lymphoma (DLBCL) treated with R-CHOP

		Overall survival		Progression-free survival		
	HR	95% CI	*P*	HR	95% CI	*P*
**DLBCL (*n* = 497)**						
IPI > 2	2.41	1.70–3.42	**< 0.001**	2.29	1.64–3.19	**< 0.001**
Female sex	1.03	0.72–1.49	0.86	0.99	0.70–1.41	0.98
Tumor size ≥ 5 cm	1.28	0.91–1.81	0.16	1.23	0.89–1.71	0.21
B-symptoms	1.35	0.94–1.94	0.099	1.31	0.93–1.85	0.12
p65^high^	1.56	0.91–2.68	0.11	1.44	0.85–2.42	0.18
**GCB-DLBCL (*n* = 243)**						
IPI > 2	2.47	1.40–4.38	**0.002**	2.39	1.39–4.09	**0.002**
Female sex	1.00	0.55–1.82	1.00	0.98	0.56–1.71	0.95
Tumor size ≥ 5 cm	1.30	0.88–1.91	0.19	1.40	0.82–2.40	0.22
B-symptoms	1.44	0.80–2.58	0.22	1.34	0.77–2.33	0.31
p65^high^	2.30	1.14–4.62	**0.02**	2.01	1.06–3.82	**0.034**
***WT-TP53* DLBCL (*n* = 338)**						
IPI > 2	2.54	1.66–3.88	**< 0.001**	2.33	1.57–3.46	**< 0.001**
Female sex	0.98	0.63–1.53	0.92	0.99	0.65–1.51	0.96
Tumor size ≥ 5 cm	1.20	0.79–1.84	0.39	1.09	0.73–1.63	0.18
B-symptoms	1.59	1.04–2.43	**0.034**	1.57	1.05–2.33	**0.028**
p65^high^	1.91	1.04–3.52	**0.037**	1.94	1.08–3.48	**0.026**

Abbreviations: R-CHOP, rituximab with cyclophosphamide, doxorubicin, vincristine, and prednisone; HR, hazard ratio; CI, confidence interval; IPI, International Prognostic Index; p65^high^, high levels of nuclear p65; GCB, germinal center B-cell–like; ABC, activated B-cell–like; WT-TP53, wild-type TP53. *Significant P values in bold.

### GEP analysis suggests different signaling pathways activated in GCB- and ABC-DLBCL

**Figure 3:**
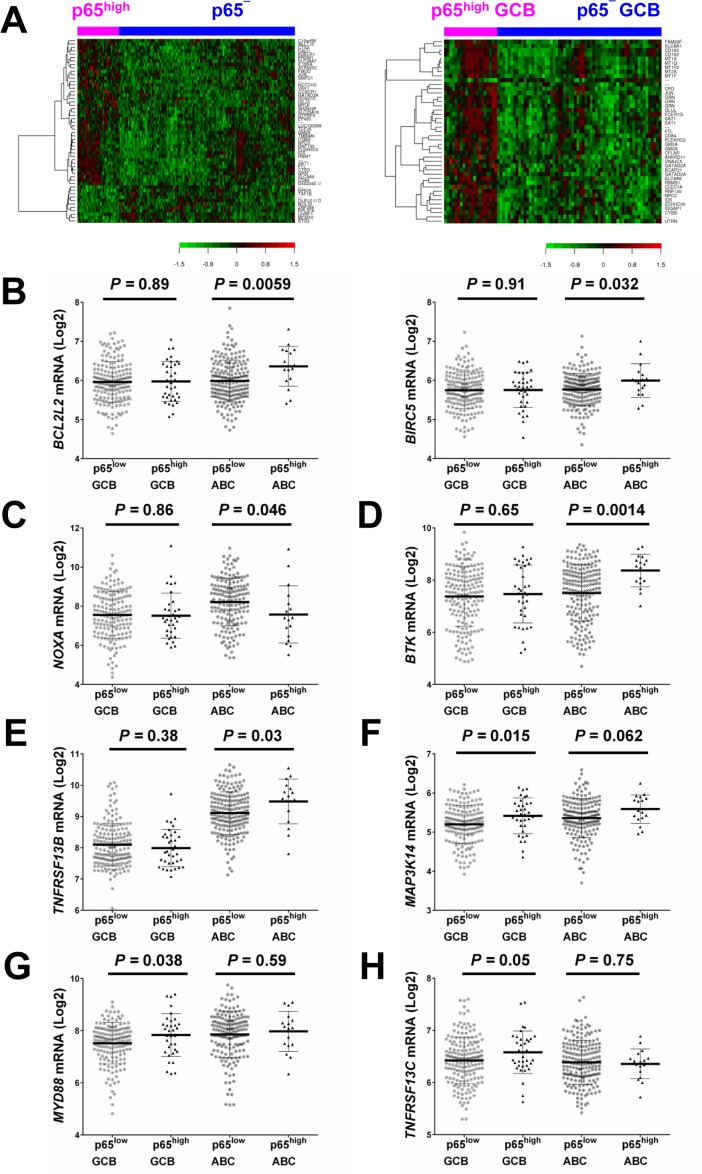
Gene expression profiling analysis (**A**) Heatmaps for comparisons between DLBCL patients with p65^high^ expression (IHC ≥50%) and those without p65 nuclear expression (IHC <10%) in the overall and GCB-DLBCL cohorts (FDR <0.15 and FDR <0.05, respectively). (**B**) *BIRC5/survivin* and *BCL2L2* were significantly upregulated in p65^high^ ABC-DLBCL. (**C**) *NOXA*/*PMAIP1* was significantly downregulated in p65^high^ ABC-DLBCL. (**D**-**E**) *BTK* and *TNFRSF13B* were significantly upregulated in the p65^high^ group in ABC-DLBCL but not in GCB-DLBCL. (**F**-**H**) *MAP3K14/NIK*, *MYD88*, and *TNFRSF13C* were significantly upregulated in the p65^high^ group in GCB-DLBCL but not in ABC-DLBCL.

**Figure 4:**
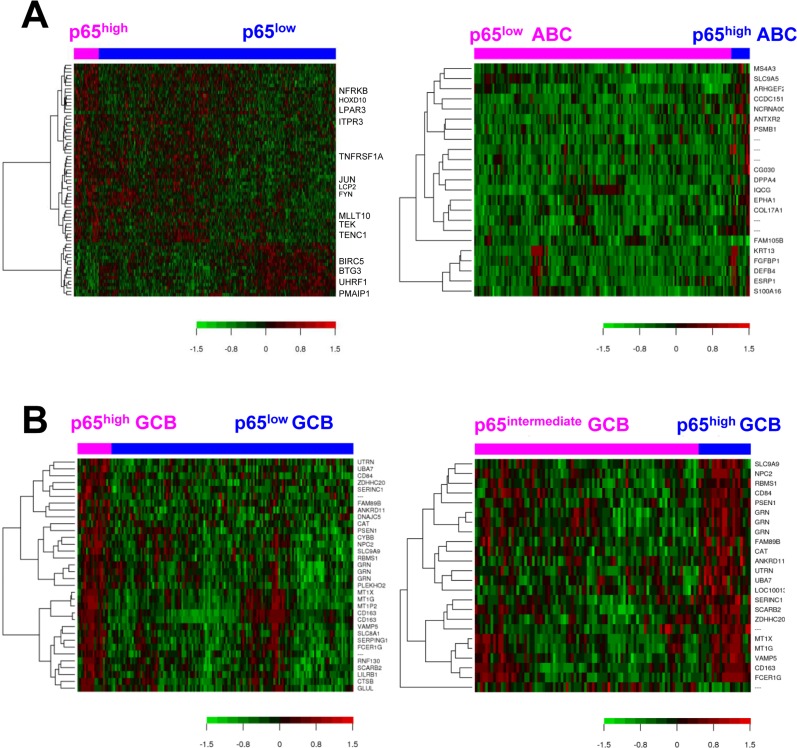
Gene expression analysis for p65 hyperactivation in diffuse large B-cell lymphoma (DLBCL) (**A**) Heatmaps for gene differentially expressed between p65^high^ (IHC ≥50%) and p65^low^ (IHC <50%) patients in DLBCL overall and in ABC-DLBCL (false discovery rate <0.30 and <0.20, respectively). (**B**) Heatmaps for genes differentially expressed between p65^high^ (IHC ≥50%) and p65^low^ (IHC <50%) patients and between p65^high^ (IHC ≥50%) and p65^intermediate^ (IHC 10-40%) patients with germinal center B-cell–like DLBCL (false discovery rate <0.05 and <0.20, respectively).

To gain insight into the molecular mechanisms underlying the prognostic effects of p65 hyperactivation in DLBCL, we compared gene expression profiles of p65^high^ and p65^low^ tumors. p65^high^ patients showed GEP signatures compared with other DLBCL including p65^−^ DLBCL patients (IHC <10%), stronger in GCB-DLBCL than in ABC-DLBCL subset (Fig. 3A, Fig. 4, Tables 3-4).

**Table 3:** Differentially expressed (canonical activation) genes between p65^high^ *vs*. p65^low^ patients with diffuse large B-cell lymphoma (DLBCL)

p65^high^ vs. p65^low^			
Functional categories	In overall DLBCL (FDR <0.30)	In GCB-DLBCL (FDR <0.05)	In ABC-DLBCL (FDR <0.20)
Signaling, ion channels	***TNFRSF1A ↑ FYN ↑ LCP2 ↑ PTPRD ↑ GTPBP2 ↑ PROCR ↑ TENC1 ↑ ITPR3 ↑ TEK ↑ CACNA2D1 ↑ AGTRAP ↑*** *LPAR3 ↓*	***MT1X ↑ MT1G ↑ SERPING1 ↑ PSEN1 ↑***	***ARHGEF2 ↑ FGFBP1 ↑ EPHA1 ↑ MS4A3 ↑***
Immune responses, inflammation		***CD163 ↑ FCER1G ↑ CYBB ↑ GRN ↑ CD84 ↑ LILRB1 ↑***	***DEFB4 ↑***
Cell cycle, DNA metabolism, transcription and translation regulation	***JUN ↑ MLLT10 ↑ GATAD2A ↑ HOXD10 ↑ NFRKB ↑*** *ZWINT ↓ MCM10 ↓ HMGB1 ↓ PPP2CA ↓UHRF1 ↓ BTG3 ↓ ZNF254 ↓ CARS ↓ PA2G4 ↓ SERBP1 ↓*	***RBMS1 ↑ ANKRD11 ↑ FAM89B ↑***	***ESRP1 ↑ DPPA4 ↑***
Apoptosis	*PMAIP1 ↓ BIRC6 ↓*	***RNF130 ↑***	
Metabolism	***SULT1A1 ↑ SPTLC2 ↑ SLC25A16 ↑ SLC9A9 ↑***	***GLUL ↑ SERINC1 ↑ CAT ↑ SLC9A9 ↑***	***S100A16 ↑ SLC9A5 ↑***
Transport, trafficking, protein folding, chaperone	***CPNE8 ↑ DNAJC5 ↑ RHD ↑ AGFG2 ↑***	***SLC8A1 ↑ NPC2 ↑ VAMP5 ↑ DNAJC5 ↑***	
Cell adhesion, cytoskeleton, collagen, extracellular matrix	***SH3D19 ↑ ITGA6 ↑ MYLK ↑ COL6A1 ↑ UTRN ↑ FMOD ↑***	***UTRN ↑***	***CCDC151 ↑ KRT13 ↑ ANTXR2 ↑ COL17A1 ↑***
Degradation, ubiquitination	***RNASE1 ↑***	***SCARB2 ↑ CTSB ↑ UBA7 ↑***	***PSMB1 ↑***
IncRNA genes, unknown function	***NCRNA00185 ↑ C19orf6 ↑ PLEKHO2 ↑ FAM124A ↑***	***MT1P2 ↑ ZDHHC20 ↑ PLEKHO2 ↑***	*FAM105B ↓* ***CG030 ↑ NCRNA00185 ↑ IQCG ↑***

Abbreviations: p65^high^, p65 immunohistochemistry results: ≥50% nuclear expression; p65^low^, p65 immunohistochemistry results: <50% nuclear expression; GCB, germinal center B-cell–like; ABC, activated B-cell–like; FDR, false discovery rate. *Upregulated genes in bold.

**Table 4:** Differentially expressed genes between p65^high^ *vs*. p65^−^ patients with diffuse large B-cell lymphoma (DLBCL) in the overall cohort and in the germinal-center-B-cell-like subgroup

Functional categories	p65^high^ vs. p65^−^ DLBCL (FDR <0.15)	p65^high^ GCB vs. p65^−^ GCB (FDR <0.05)
Signaling, ion channels	***IL10RA ↑ GPS2 /// D4S234E ↑ NOTCH3 ↑ VAV1 ↑***	***FAM26F ↑ MT1X ↑ MT1G ↑ MT2A ↑ MT1F ↑ CFLAR ↑ CLEC7A ↑***
Immune responses, inflammation	***GRN ↑***	***CD163 ↑ GRN ↑ FCER1G ↑ CD84 ↑ CYBB ↑***
Cell cycle, DNA metabolism, transcription and translation regulation	***JUN ↑ PRDM1 ↑ GATAD2A ↑ TCF25 ↑ MLLT10 ↑ EP400 ↑ HOXD10 ↑*** *TAF1B ↓ ZNF254 ↓ RPL37A ↓ UHRF1 ↓ MCM10 ↓ BTG3 ↓*	***JUN ↑ GATAD2A ↑ RBMS1 ↑***
Apoptosis	***TMBIM6 ↑ RNF130 ↑*** *BIRC6 ↓*	***RNF130 ↑***
Metabolism, redox regulation	***SLC9A9 ↑ ATP6V0C ↑ SLC25A16 ↑ SAT1 ↑ SMPD1 ↑ C10orf58 ↑ HNMT ↑ GTPBP2 ↑***	***CPD ↑ GLUL ↑ SAT1 ↑ FTL ↑ ANKRD11 ↑ SLC9A9 ↑***
Transport, trafficking, protein folding, modification	***NPC2 ↑ WASH3P ↑ FTL ↑ GM2A ↑ KDELR1 ↑ SLC39A7 ↑ CTSD ↑ CALU ↑***	***SLC8A1 ↑ GM2A ↑ DNAJC5 ↑ BCAP31 ↑ NPC2 ↑***
Cell adhesion, cytoskeleton, collagen, extracellular matrix	***ITGB2 ↑ MYLK ↑ CD84 ↑ FMOD ↑***	***IQGAP1 ↑ UTRN ↑***
Degradation	***CTSZ ↑ IDS ↑***	***IDS ↑***
IncRNA genes, other function	***PLEKHO2 ↑LOC100288142 /// NBPF1 /// NBPF10 ↑ CCHCR1 ↑ IGLJ3 ↑*** *DLEU2 /// DLEU2L ↓ NOL10 ↓*	***MT1P2 ↑ PLEKHO2 ↑ ZDHHC20 ↑***

Abbreviations: p65^high^, high p65 nuclear expression (immunohistochemistry results: ≥50%); p65^−^, negative p65 nuclear expression (immunohistochemistry results: <10%); FDR, false discovery rate. *Upregulated genes in bold.

In line with the unfavorable prognosis of patients with p65^high^ DLBCL, GEP analysis found that *JUN* and *PTPRD* (involved in cell cycle progression) were upregulated (1.43-fold and 1.31-fold respectively) whereas pro-apoptotic *NOXA*/*PMAIP1* and *BTG3* which negatively regulates proliferation and cell cycle progression were downregulated (1.62-fold and 1.45-fold, respectively) in p65^high^ DLBCL compared with p65^low^ DLBCL. *RBMS1* which transactivates *MYC* was upregulated (1.48-fold) in p65^high^ compared with p65^low^ GCB-DLBCL (Table 3). Paradoxically, antiapoptotic *BIRC6*, *MCM10* (involved in the initiation of eukaryotic genome replication), *CARS* (cysteinyl-tRNA synthetase) and *PA2G4* (involved in growth regulation) were downregulated in p65^high^ patients, and *TENC1* (which inhibits AKT1 signaling) was upregulated in p65^high^ DLBCL.

When analyzed in GCB and ABC subtypes individually, we found such paradoxical association was limited in the GCB subset. *RNF130* involved in apoptosis showed 1.81-fold upregulation in p65^high^ GCB-DLBCL patients. In contrast, in p65^high^ ABC-DLBCL, antiapoptotic *BIRC5* and *BCL2L2* were significantly upregulated whereas pro-apoptotic *NOXA*/*PMAIP1* was significantly downregulated (Fig. 3B-C), in addition to the proliferative signatures (such as upregulation of genes involved in replication, transcription, translation, and metabolism) in ABC-DLBCL (Table 3).

GEP suggested that in GCB-DLBCL, instead of antiapoptotic mechanisms, dysregulations in immune responses and tumor microenvironment may be relevant for the poor prognosis associated with p65^high^. Such immune signatures included *FCER1G* (Fc fragment of IgE high affinity I receptor for gamma subunit), 2.21-fold upregulation, *CYBB* (critical component of phagocytes generating superoxide), 1.77-fold upregulation, granulin gene *GRN*, 1.63-fold up-regulation, *LILRB1* (a MHC class I receptor resulting in immunosuppression), 1.49-fold upregulation, *CD163* (an antigen exclusively expressed in monocytes and macrophages), 2.46-fold upregulation, and *CD84* (an adhesion molecule involved in regulating receptor-mediated signaling in immune cells), 1.55-fold up-regulation. In the GEP comparison in overall DLBCL, a few immune-related genes were also found up- or down-regulated in p65^high^ DLBCL compared with p65^low^ DLBCL, including upregulation of *LCP2* (lymphocyte cytosolic protein 2, involved in T cell receptor signaling, 1.27-fold) and *TEK* (anti-inflammatory, 1.21-fold), and downregulation of *UHRF1* (an epigenetic regulator promoting proliferation of immunosuppressive Treg cell, 1.48-fold down-regulation) [43] in p65^high^ DLBCL (Table 3).

These data indicated that the antiapoptotic and pro-proliferation function of p65 was primarily activated in ABC-DLBCL, whereas immune dysregulation might be more relevant for the significantly adverse impact of p65 hyperactivation in GCB-DLBCL. We further analyzed the expression of p65-activating upstream signals in GCB- and ABC-DLBCL. *TNFRSF1A* en-coding a TNF-α receptor which can activate NF-κB by degrading inhibitory IkBα (canonical activation), was upregulated in the overall p65^high^ group than in the overall p65^low^ group (*P* = 0.0001). *LPAR3* which encodes a receptor for lyso-phosphatidic acid/LPA was downregulated in p65^high^ DLBCL. In the ABC-DLBCL subset only, *PSMB1*, which encodes a 20S core beta subunit of the proteasome B-type family, was upregulated in p65^high^ compared with p65^low^ patients (suggesting canonical activation of NF-kB). Bruton tyrosine kinase gene BTK which plays an important role in BCR signaling activation (canonical activation), and *TNFRSF13B* which encodes the tumor necrosis factor receptor for APRIL and BAFF were significantly upregulated in p65^high^ ABC-DLBCL but not in p65^high^ GCB-DLBCL (Fig. 3D-E). In comparison, in GCB-DLBCL but not in ABC-DLBCL, *TNFRSF13C* which encodes the receptor specific for BAFF (non-canonical activation), *MYD88* which encodes an adapter protein essential for the Toll-like receptor (TLR) and interleukin-1 receptor signaling pathways, and *MAP3K14*/*NIK* which is involved in non-canonical activation of NF-κB were significantly upregulated in the p65^high^ compared with p65^low^ group (Fig. 3F-H).

### Targeting NF-κB in DLBCL cells

#### Molecular inhibition of constitutive NF-κB activation in DLBCL cell lines

**Figure 5:**
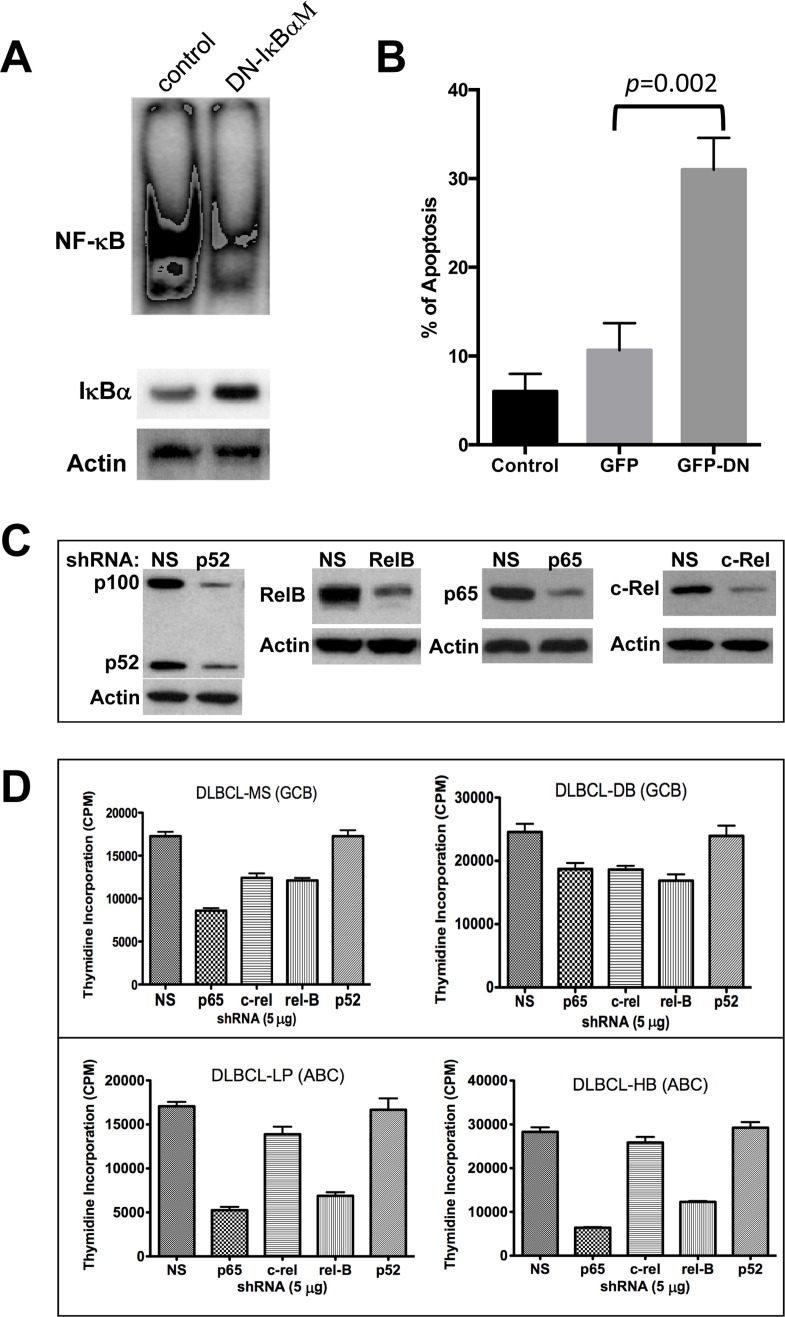
Molecular targeting of NF-03BA;B in diffuse large B-cell lymphoma (DLBCL) cell lines (**A**) DLBCL-MS cells were transfected with empty control vector or a pCMV-IκBαM vector for 24 hrs. Nuclear extracts (10 μg) were analyzed for NF-κB expression by EMSA. Cytoplasmic extracts were assessed for IκBα and actin protein expression by Western blotting. (**B**) Transfected cells from part **A** were also assessed for apoptosis after 24 hours of incubation using annexin V assays. (**C**) MS cells were transfected with plasmids expressing the p52, RelB, p65, c-Rel, or a non-specific (NS) shRNA. Forty-eight hours post-transfection, proteins were extracted and analyzed for NF-κB component inhibition by Western blot. (**D**) Indicated DLBCL cell lines were transfected with the validated green fluorescent protein (GFP)-plasmid–based shRNA for each of the NF-κB subunits. After 16 hours, GFP–positive cells were sorted and assessed using proliferation assays. Data represent two independent experiments with triplicate samples. **Abbreviations**: GCB, germinal center B-cell–like; ABC, activated B-cell–like, DN, dominant negative.

First, we examined whether specific inhibition of NF-κB was sufficient to block cell survival by over-expressing a super repressor mutant form of IκBα (pCMV-IκBαM) in a representative DLBCL cell line, MS, that has been previously shown to have constitutive NF-κB activation [44]. IκBαM binds to NF-κB subunits but cannot be phosphorylated on the basis of alanine substitution for serines 32 and 36, acting as a dominant negative (DN) and thereby preventing the NF-κB subunits from translocating into the nucleus. Transient transfection (70-80 efficiency and 75% viability) of a DLBCL-MS cell line with the DN-IκBαM leads to the induction of IκBα protein level while suppressing constitutive NF-κB activation (Fig. 5A). In addition, cells over-expressing the DN-IκBαM are prone to apoptosis as demonstrated by Annexin V binding assays (Fig. 5B). This result suggests that constitutive NF-κB activation is required for the survival of this cell line. To determine the functional significance of each NF-κB subunit on growth and survival regulation in DLBCL, we used specific validated shRNAs to selectively silence each NF-κB component individually in four representative DLBCL cell lines (two GCB-DLBCLs, two ABC-DLBCLs). These validated shRNAs inhibited endogenous NF-κB by more than 70% (Fig. 5C). Except for p52, downregulation of p65, c-Rel, and RelB protein expression with individual shRNAs inhibited DLBCL cell growth (thymidine incorporation assay), and inhibition of p65 was most effective (Fig. 5D), particularly in cell lines with mutated p53 (MS, LP and HB).

### Pharmacological targeting of constitutive NF-κB activation in DLBCL cells

To evaluate the effects of pharmacological inhibition of NF-κB activation on transcription activities of NF-κB subunits and DLBCL cell growth and survival, we selected the proteasome inhibitor bortezomib (BZ), and the small molecule NF-κB inhibitor BAY 11-7082 (BAY-11) that selectively inhibits the phosphorylation and degradation of IκBɑ[45-47] in MS (GCB-DLBCL) cells.

**Figure 6:**
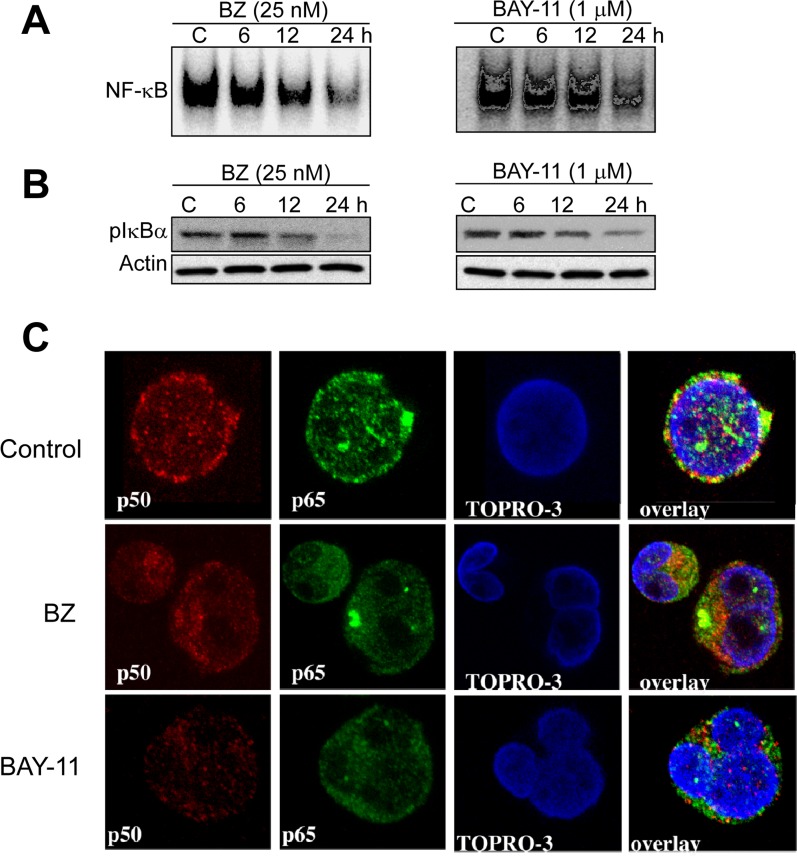
Pharmacological inhibition of constitutive NF-03BA;B activation in DLBCL cells (**A-B**) DLBCL cells (MS) were cultured in the presence of bortezomib (BZ, 25 nM) or BAY-11 (1 μM) for the indicated time points (hours). Nuclear extracts were purified and subjected to EMSA analyzed for NF-κB DNA binding activity; cytoplasmic extracts were subjected to immunobloting for pIκBa and actin. (**C**) DLBCL-MS cells cultured in the presence of bortezomib (BZ, 25 nM) or BAY-11 (1 μM) for 24 hours and then analyzed for p50 (red) and p65 (green) protein expression by confocal microscopy analysis. Topro-3 (blue) serves as a nuclear staining marker.

**Figure 7:**
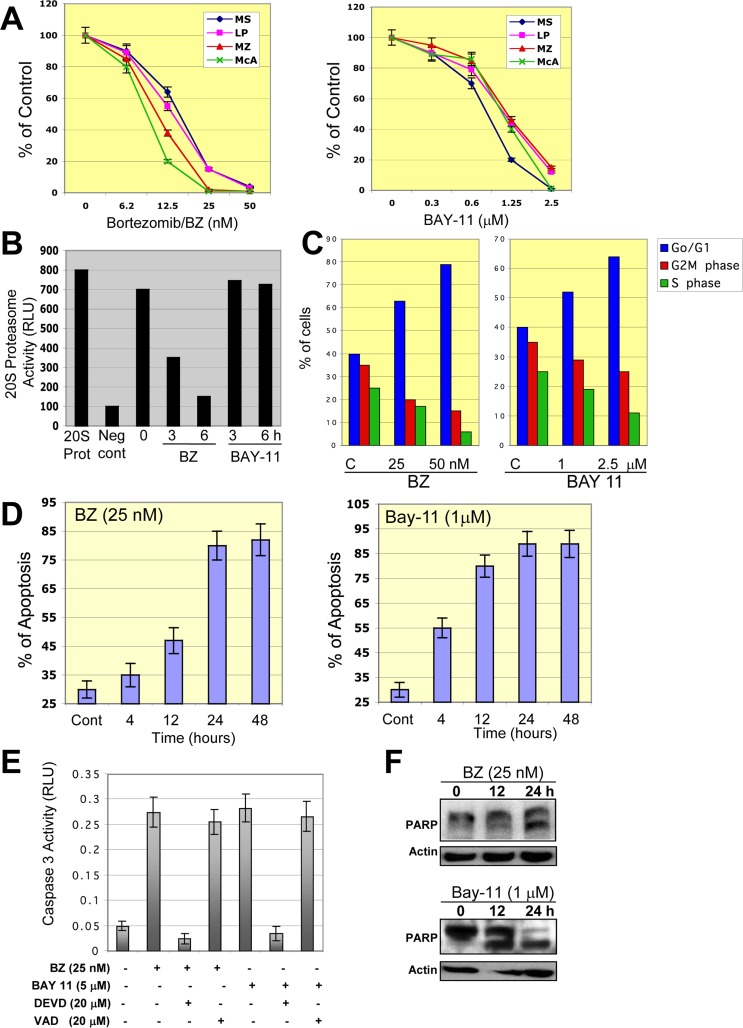
Inhibition of NF-03BA;B in DLBCL cells leads to cell growth inhibition, G0/G1 cell cycle arrest, and apoptosis (**A**) Representative ABC- and GCB-DLBCL cell lines were treated with bortezomib (BZ) or BAY-11 for 48 hours and cell proliferation was measured using 3H-thymidine incorporation assays. The percentages of growth inhibition of treated cells relative to untreated (control cells) were plotted. The data shown are the means and ranges of triplicate cultures from three independent experiments. (**B**) DLBCL-MS cells were cultured in the absence or presence of bortezomib (BZ) or BAY-11 and subjected to a 20S proteasome assay. Purified 20S proteasome was used as a positive control. **Abbreviations**: RLU, relative light unit; 20S pro, 20S proteasome, Neg Cont., negative control. (**C**) DLBCL-MS cells were cultured in the absence or presence of BZ (50 nM) or BAY-11 (1 μM) and analyzed for cell cycle profile. The percentages of cells in G0/G1, S, and G2M phases are shown. (**D**) DLBCL-MS cells were cultured in the absence or presence of BZ (50 nM) or BAY-11 (1 μM) for the indicated time points and then analyzed for apoptosis using annexin V assays. (**E**) DLBCL-MS cells were cultured in the presence of BZ (50 nM) or BAY-11 (1 μM) and in some cases with the caspase 3 inhibitor DEVD or the caspse 1 inhibitor VAD. Caspase 3 activity was measured after 24 hours of treatments. Caspase 3 activity was observed after 12 hours of treatment. **Abbreviations**: RLU, relative light units. (**F**) DLBCL-MS cells were cultured in the presence of BZ (50 nM) or BAY-11 (1 μM) for the indicated time points and cell extracts were subjected to Western blotting for a known caspase substrate, poly-(ADP-ribose) polymerase (PARP) cleavage.

To ascertain whether BZ or BAY-11 has an effect on constitutive NF-κB activation in DLBCL cells, we performed EMSA with nuclear extracts purified from BZ- or BAY-11-treated GCB-DLBCL cell line (MS). After BZ or BAY-11 treatment, NF-κB DNA-binding activity (Fig. 6A) and the level of phosphorylated-IκBɑ (Fig. 6B) gradually declined in a dose-dependent manner in the MS DLBCL cell line. Confocal microscopy analysis also demonstrated that BZ or BAY-11 treatment inhibits the nuclear accumulation of p50 and p65 NF-κB subunits, leading to DNA fragmentation, indicative of cells undergoing apoptosis (Fig. 6C). Next, we evaluated the effects of BZ or BAY-11 on DLBCL cell viability using *in vitro* proliferation assays in four representative DLBCL cell lines (two ABC, two GCB). Both BZ and BAY-11 inhibited cell proliferation in the representative DLBC cell lines in a dose-dependent manner (Fig. 7A). BZ and BAY-11 inhibit NF-κB by different mechanisms, as we analyzed the cell lysates from BZ-treated and BAY-11-treated DLBCL-MS cells to a 20S proteasome proteolysis assay, and found proteasome activity was substantially inhibited (>50%) after 3 hours of BZ treatment, whereas BAY-11 treatment did not affect proteasome activity in a similar time points (Fig. 7B). To determine whether the cell growth inhibition effects of BZ and BAY-11 involve their activity in the cell cycle regulation, we analyzed the cell cycle profile. As shown in Fig. 7C, in a representative DLBCL cell line (MS), both BZ-and BAY-11-treated DLBCL cells accumulated in the G0/G1 phase of the cell cycle, while cells in G2M and S phases were decreased. In addition, BZ or BAY-11 treatments in MS DLBCL cell line resulting in cells undergoing apoptosis in a time-dependent manner (Fig. 7D). To verify that these cells had actually undergone apoptosis, we measured the generation of caspase 3 activities. DLBCL cells treated with BZ or BAY-11 activated caspase 3 activity after 12 hours of treatment, which can be block with a caspase 3 inhibitor (DVED) but not with a caspase 1 inhibitor (VAD) (Fig. 7E). In addition, a known caspase substrate, poly-(ADP-ribose) polymerase (PARP), was cleaved after BZ or BAY-11 treatment (Fig. 7F). These experiments provide additional and interesting insights into the putative role of NF-κB in DLBCL cell proliferation and viability maintenance.

## DISCUSSION

In this study we studied the significance of RelA/p65 NF-κB in DLBCL by two ways. In the first part of this study, we evaluated the prognostic significance of RelA/p65 nuclear expression in a large cohort of *de novo* DLBCL treated with R-CHOP (*n* = 487). Although p65 nuclear expression may not be a strong prognostic marker in overall DLBCL, we found p65 hyperactivation (IHC ≥50%) had significant adverse impact on survival of patients with stage I/II DLBCL independent of cell-of-origin and *TP53* mutation status, even though it was not associated with apparent genetic or phenotypic abnormalities, such as *TP53* mutations, *MYC*/*BCL2* translocation, and Myc/Bcl-2 overexpression.

The adverse prognostic significance of p65 hyperactivation was also seen in the GCB-DLBCL subtype overall and in the subset with wild-type *TP53*, but not in the subsets with strong unfavorable factors including advanced stages, *TP53* mutations, and ABC cell-of-origin. In addition, lower levels (10-40%) of p65 nuclear expression did not have significant prognostic impact in DLBCL. This limited significance of p65 expression in DLBCL may reflect different signaling transductions pathways activating p65, different p65 NF-κB dimers, and complicated p65 functions influenced by other factors including p53 in different stimulatory signals. For example, NF-κB p65 activation induced by cytotoxic stimuli promotes apoptosis in mouse embryo fibroblasts, which contrasts with the prosurvival function of p65 induced by inflammatory cytokines [30]. In various cancer cell lines, p65 and p53 formed p65/p53 complex and bound to DNA targets; the function of p65 and fate of tumor cells are significantly affected by p53 and stress levels [32]. Others have shown that there is transcriptional and functional crosstalk between NF-κB and p53. p53 can negatively regulate NF-κB activation by regulating *IKK1* expression [29] and suppressing glycolysis [28]; NF-κB and p53 antagonize each other's function in apoptosis, proliferation and tumor invasion that appears to depend on cellular context. Overall the results in this study suggested that p65 and wild-type p53 counter-acted each other in DLBCL, and that the inhibition of p53 tumor suppressor function by p65 hyperactivation had a significant adverse impact on clinical outcomes.

GEP suggested that BCR, TNF, TLR, and mitogen-activated protein kinase signaling pathways were all implicated in p65 hyperactivation in DLBCL. These upstream pathways were activated preferably in ABC-DLBCL or GCB-DLBCL, and correspondingly, resulting in different downstream pathways in ABC and GCB subtypes. In ABC-DLBCL, p65^high^ GEP signatures were featured by proliferation and anti-apoptosis, whereas in GCB-DLBCL in which subgroup p65 hyperactivation showed significant adverse prognostic impact, p65^high^ expression was accompanied with upregulation of some pro-apoptosis genes as well as many immune genes. Upregulation of *LILRB1* and *CD163* in p65^high^ patients suggested immune suppres-sion and dysregulation, which may contribute to the associated poor prognosis.

In the second part of this study, we tested whether NF-κB p65 subunit in particular is a potential molecular target by the proteasome inhibitor bortezomib and the small molecule NF-κB inhibitor BAY-11 *in vitro*. Previous studies have shown that proteasome inhibitors have better antitumor efficacy in patients with ABC-DLBCL than in patients with GCB-DLBCL, probably due to higher p65 expression in the ABC subtype [45, 48, 52]. Consistently, our GEP analysis also found the proteasome gene *PSMB1* was upregulated in ABC-DLBCL. Our *in vitro* experiments found that these anti-NF-κB agents can effectively inhibit p65 protein expression and DNA binding activity, leading to cell cycle arrest, decreased cell proliferation, and apoptosis induction in both GCB and ABC types of p65-overexpressing DLBCL cell lines. Intriguingly, representative DLBCL cell lines with mutated p53 are more sensitive to p65 shRNA targeting approach as compared to a cell line with wild-type p53, opposite the prognostic effects observed in the DLBCL study cohort. These findings may suggested that although p65 subunit only manifested prognostic significance in certain DLBCL subsets due to the complexity of NF-κB dimers and activating mechanisms, *in vitro* experiments nonetheless demonstrated that NF-κB overexpressing DLBCL cells were addictive to NF-κB and vulnerable for NF-κB inhibitors. This vulnerability of DLBCL cells was also apparent in the context of mutated p53; p65 may have an important role in the oncogenic activities of mutated p53 in DLBCL. Importantly, *in vitro* p65 subunit stood out as a critical factor in controlling cell growth and survival and showed the most sensitivity to molecular and pharmacological inhibition of NF-κB activation. Therefore, our current study in both patient samples and DLBCL cell lines provided additional insights into the putative roles of NF-κB p65 in immune regulation, DLBCL cell proliferation, and viability maintenance, and the utility of p65 as a biomarker to stratify DLBCL patients to receive alternative therapeutic regimens including agents targeting NF-κB [52]. However, these findings warrant further investigation and validation in more representative DLBCL cell lines as well as primary DLBCL cells.

In summary, we provide clinical and experimental data that RelA/p65 NF-κB has prognostic and therapeutic value in DLBCL. High p65 nuclear expression is a significant adverse biomarker in patients with early-stage (I/II) DLBCL. Pharmacological p65 inactivation effectively inhibited cell growth and survival in both GCB-DLBCL and ABC-DLBCL cell lines with p65 hyperactivation.

## METHODS

### Patients

The study cohort included 487 patients with *de novo* DLBCL treated with R-CHOP, as part of the International DLBCL R-CHOP Consortium Program. All patients were diagnosed as DLBCL between 2001 and 2012 according to the World Health Organization classification criteria, and did not have a history of low-grade B-cell lymphoma, primary mediastinal, cutaneous, central nervous system DLBCL, or human immunodeficiency virus infection. Informed consent was obtained from all patients. This study was conducted in accordance with the Helsinki Declaration and was approved by the Institutional Review Boards of all participating centers. GCB/ABC subtype classification by GEP or immuno-histochemistry algorithms [49], and *TP53* mutation detection using p53 AmpliChip [39] have been described previously. Overall survival (OS) was calculated from the date of diagnosis to the date of death from any cause or the date of last follow-up for censored patients. Progression-free survival (PFS) was calculated from the date of diagnosis to the date of disease progression, recurrence, or patient death from any cause. Survival analysis was performed using the Kaplan-Meier method and the log-rank (Mantel-Cox) test. The clinical features of DLBCL patients with high or low levels of p65 at the time of presentation were compared using the chi-square test. Univariate survival analysis was performed using the GraphPad Prism 6 (GraphPad Software, San Diego, CA). Multivariate survival analysis was performed using the Cox regression model and SPSS software (version 19.0; IBM Corporation, Armonk, NY). *P* values ≤0.05 were considered statistically significant.

### Immunohistochemical staining

Immunohistochemistry for p65 and other NF-κB subunits using specific antibodies (Abcam, Cambridge, MA) was performed on tissue microarrays of formalin-fixed, paraffin-embedded lymphoma samples using methods described previously [10, 49]. The immuno-histochemical stains were assessed in 10% increments by three pathologists blinded to the clinical outcomes. Disagreements about the percentage of positive cells were resolved by joint review at a multi-headed microscope.

### Gene expression profiling

GEP was performed using the Affymetrix GeneChip Human Genome U133 Plus 2.0 array (Santa Clara, CA) and CEL files were deposited in the NCBI Gene Expression Omnibus repository (GSE#31312) [49]. GEP were available for 444 DLBCL patients of this study cohort with high or low levels of p65 nuclear expression. The *P* values for differential expression obtained via multiple *t*-tests were corrected for false discovery rates using the beta-uniform mixture method.

### *In vitro* studies

#### Cell lines

Human DLBCL cell lines MS (mutated *TP53*) and DB (wild-type for p53) (GCB subtype), as well as LP (mutated *TP53*) and HB (mutated *TP53*) (ABC subtype) were previously characterized and described [44, 50]. The DLBCL cells were cultured in Roswell Park Memo-rial Institute medium (Life Technologies, Carlsbad, CA) containing 15% fetal calf serum and 1% penicillin/streptomycin (HyClone Laboratories, Logan, UT).

#### Antibodies and small hairpin RNA plasmids

The following primary antibodies were used: p50, p65, c-Rel and p52 (Millipore, Billerica, MA), and RelB (Santa Cruz Biotechnology, Santa Cruz, CA). The SureSilencing small hairpin RNA (shRNA) green fluorescent protein (GFP)–based plasmids for the NF-κB subunits p52, p65, c-Rel, and RelB were purchased from SuperArray Biosciences (Frederick, MD). pCMV-IκBαM and control vectors were purchased from Clontech Laboratories (Mountain View, CA).

#### Transfection

Transfection experiments in DLBCL cells with validated green fluorescent protein (GFP)-shRNAs were performed *in vitro* in representative transfectable DLBCL cells, using the Neon transfection system from Invitrogen (Life Technologies Corporation, Grand Island, NY) as described previously [44], and were repeated at least twice to verify reproducible experimental results. Twenty-four hours after transfection, GFP^+^ cells were sorted by a fluorescence-activated cell sorter (FACS) flow cytometer and plated. Cell proliferation was measured 96 hours after sorting by thymidine incorporation assays, while some cells were lysed for Western blot analysis for NF-κB subunit inhibition. A set of four shRNA plasmids for each NF-κB subunit was tested and the optimal (>75%) gene knock-down shRNA plasmid was selected.

#### Therapeutic NF-κB inhibition experiment

DLBCL cells were treated with increasing doses of bortezomib (0–50 nM), or organic compounds BAY 11-7082/BAY-11 (1μM) for 6-48 hours and subjected to cell proliferation assays, electromobility gel shift assay (EMSA), immunofluorescence, apoptosis detection assay, and cell cycle analysis according to the manufacturer's instructions or procedures as previously described [51]. Data are representative of three independent experiments.

#### Thymidine incorporation assays

In vitro thymidine incorporation proliferation assays were performed as described previously ^1^. Briefly, cells were plated (in triplicate) at 4.0 × 10^4^ cells/well in 200 μl of RPMI 1640 with 10% FCS and the indicated reagents in a 96-well plate and incubated in 5% CO_2_ at 37°C. After 72 h, each well was pulsed with 0.5 μCi/10 μl of [^3^H]thymidine (Amersham, Arlington Heights, IL) for 16 h. Cells were harvested and the radioactivity was measured.
